# The middle cerebral artery (MCA) dot sign

**DOI:** 10.1002/ccr3.3319

**Published:** 2021-08-19

**Authors:** Mohamad Syafeeq Faeez Md Noh

**Affiliations:** ^1^ Department of Imaging Level 3, Faculty of Medicine and Health Sciences Universiti Putra Malaysia Serdang Malaysia; ^2^ Department of Radiology Universiti Putra Malaysia (UPM) Teaching Hospital Persiaran MARDI‐UPM Serdang Malaysia

**Keywords:** acute ischemic stroke (AIS), computed tomography (CT), middle cerebral artery (MCA)

## Abstract

The middle cerebral artery (MCA) dot sign is an important radiological sign in patients presenting with acute ischemic stroke (AIS). If identified and intervened early, a good clinical outcome may be achieved

## Graphical abstract

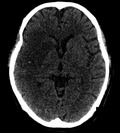

## 1 QUESTION

What is the MCA dot sign and its significance?

## 2 CASE SUMMARY

**FIGURE 1:**
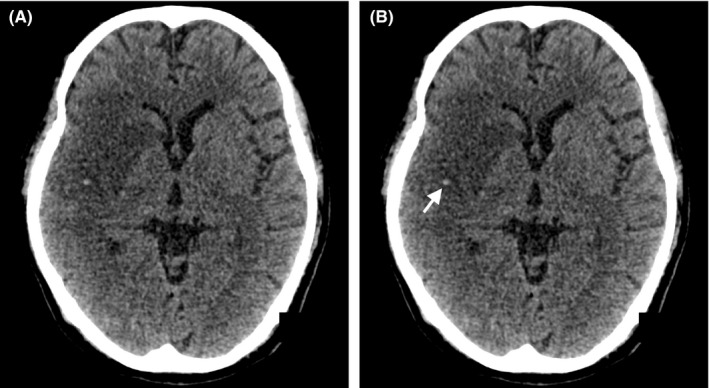
A, Nonenhanced cranial CT showing right‐sided MCA territory infarct. B, A hyperdense punctate focus (arrow) is noted in the right Sylvian fissure, corresponding to the MCA dot sign

A 55‐year‐old man, with underlying diabetes and hypertension, was referred from an outside center to us with a history of acute left‐sided body weakness, facial asymmetry, and slurred speech for more than 24 hours. On examination, left‐sided hemiplegia was noted. The sensation on the same side was markedly reduced, with brisk reflexes of both the ipsilateral upper limb (biceps, triceps, supinator, finger jerks) and lower limb (patellar, ankle jerks), as well as hypertonia. A nonenhanced cranial computed tomography (CT) showed a right middle cerebral artery (MCA) dot sign (Figure 1), with an established infarct. In view of the clinical and radiological findings, the family was counseled and decided to not pursue thrombolysis and mechanical thrombectomy. He was referred to the stroke unit for medical management (antiplatelet (aspirin), statin, and antihypertensive therapy) and rehabilitation.

## 3 ANSWER

The MCA dot sign is a radiological sign representing a thromboembolus within a segmental branch of the MCA located within the Sylvian fissure (M2 or M3 segment). One study of 100 patients noted a higher detection rate of the MCA dot sign (16%) when compared to the more familiar, hyperdense MCA sign (5%), in patients with AIS presenting within 3 hours of symptom onset.^1^ Detection of this sign correlates with angiographically confirmed M2 or M3 branch vessel clot, with sensitivity of 38%, specificity of 100%, positive predictive value of 100%, and negative predictive value of 68%.^2^ Due to the distal location (and smaller area of infarct), patients with this sign, when intervened early, fare better compared to those with findings of the hyperdense MCA sign.

## CONFLICT OF INTEREST

None declared.

## AUTHOR CONTRIBUTIONS

MSF: is responsible for the care of the patient, literature review, and preparation of this manuscript.

## ETHICAL APPROVAL

The author's institution does not require ethical approval for the publication of single case studies.

## INFORMED CONSENT

Informed consent obtained from patient.
